# Single-cell RNA sequencing explores the evolution of the ecosystem from leukoplakia to head and neck squamous cell carcinoma

**DOI:** 10.1038/s41598-024-58978-9

**Published:** 2024-04-06

**Authors:** Haibin Wang, Zhenjie Guan, Lian Zheng

**Affiliations:** 1 Department of Oral and Maxillofacial Surgery, The First Affiliated Hospital of Zhengzhou University, Zhengzhou, 450052 China; 2 Department of Stomatology, The First Affiliated Hospital of Zhengzhou University, Zhengzhou, 450052 China

**Keywords:** Head and neck squamous cell carcinoma, Leukoplakia, Ecosystem evolution, Differentiation trajectory, Prognosis, Head and neck cancer, Oral cancer, Oral cancer

## Abstract

It has been found that progression from leukoplakia to head and neck squamous cell carcinoma (HNSCC) is a long-term process that may involve changes in the multicellular ecosystem. We acquired scRNA-seq samples information from gene expression omnibus and UCSC Xena database. The BEAM function was used to construct the pseudotime trajectory and analyze the differentially expressed genes in different branches. We used the ssGSEA method to explore the correlation between each cell subgroup and survival time, and obtained the cell subgroup related to prognosis. During the progression from leukoplakia to HNSCC, we found several prognostic cell subgroups, such as AURKB + epithelial cells, SFRP1 + fibroblasts, SLC7A8 + macrophages, FCER1A + CD1C + dendritic cells, and TRGC2 + NK/T cells. All cell subgroups had two different fates, one tending to cell proliferation, migration, and enhancement of angiogenesis capacity, and the other tending to inflammatory immune response, leukocyte chemotaxis, and T cell activation. Tumor-promoting genes such as CD163 and CD209 were highly expressed in the myeloid cells, and depletion marker genes such as TIGIT, LAG3 were highly expressed in NK/T cells. Our study may provide a reference for the molecular mechanism of HNSCC and theoretical basis for the development of new therapeutic strategies.

## Introduction

Head and neck squamous cell carcinoma (HNSCC) is the most common type of head and neck cancer, usually occurring in the oral cavity, pharynx, larynx, nasal cavity and other epithelial tissues, with high aggressiveness and mortality^1^. Despite advances in the treatment of HNSCC, HNSCC is the eighth most common cancer worldwide and still causes more than 400,000 deaths each year^2,3^. The pathogenesis of HNSCC is complex, and the known risk factors include smoking, drinking, HPV infection and so on^4^. HPV infection may be an important cause of HNSCC, so many studies often distinguish HNSCC as HPV-negative or HPV-positive^5^.

Most patients with HNSCC are diagnosed at a locally advanced stage, where large local lesions and distal metastases may occur, requiring a combination of therapies^6^. The traditional methods of HNSCC are mainly surgery and radiotherapy, supplemented by chemotherapy and immunotherapy^7^. At present, immunotherapy such as programmed death 1 (PD-1) inhibitors and molecular therapy such as cetuximab are also relatively effective therapies for HNSCC, and single or combined drugs can be used to improve the treatment strategy^8^. HNSCC is highly invasive and heterogeneous. After targeted treatment, the prognosis of some advanced patients is still not optimistic, and there may be recurrence and metastasis^9^.

The evolution of HNSCC is a long-term process, usually progressing from normal epithelial tissue to leukoplakia, and then further worsening to primary HNSCC, and finally, distal metastasis may occur^10^. Malignant transformation of leukoplakia is an important factor leading to HNSCC, and the lifetime malignant conversion rate of leukoplakia patients is about 20%^11^. The evolution of leukoplakia to HNSCC involves changes in genetic landscape, histomathological features and immune landscape^12^. Changes in metabolic pathways, including lipid metabolism, amino acid metabolism and glycolysis, can be found when detecting mucosal secretions in HNSCC patients^13^. However, the specific process and mechanism of how leukoplakia is transformed into HNSCC are still insufficient.

In this study, based on HPV-negative leucoplakia samples and HNSCC samples, the heterogeneity of epithelial cells, fibroblasts, myeloid cells and NK/T cells was analyzed sequentially by single-cell RNA sequencing (scRNA-seq) technology, and the biological processes involved in each cell subgroup were explored. Then, the differentiation trajectories of these cells from leukoplakia to HNSCC were constructed in order to explore the ecosystem changes from leukoplakia to HNSCC. We hope that we could provide new ideas for the molecular mechanism of HNSCC occurrence and development, and help improve the treatment strategy of HNSCC.

## Material and methods

### Download of scRNA-seq data

We download the GSE181919 dataset from the Gene Expression Omnibus (GEO)^14^. In order to avoid the influence of HPV infection on the occurrence and development of HNSCC, we only retain HPV-negative samples. The sampling sites included oral cavity, hypopharynx, oropharynx. The single-cell sequencer is 10 × Genomics, Barcoded sequencing libraries were generated using Chromium Single Cell 3′ v2 Reagent Kits and sequenced using the HiSeq 4000 platform (Illumina).

### Download of TCGA-HNSCC expression profile

We downloaded RNA-seq data of TCGA-HNSCC from UCSC Xena database with expression value log2(fpkm + 1)^15,16^, and downloaded the total survival time and survival status. We removed the samples with a total survival of less than 90 days, leaving 476.

### Filtering, standardization, dimensionality reduction and clustering of scRNA-seq data

We used the Seurat package to read the gene expression matrix, keeping cells with a detected number of genes between 200 and 8000 and a proportion of mitochondrial genes less than 10%^17^. Remove genes that are expressed in less than 0.1% of cells. We used SCTransform function to normalize, after PCA dimensionality reduction, harmony package to remove batch effect between samples, and RunUMAP function (dims = 1:30) to reduce dimensionality^18,19^. Finally, the FindNeighbors and FindClusters functions are used to cluster the cell subgroups^20^ (Fig. S1). For all cell clusters, dims = 1:30, resolution = 0.1. For epithelial cells, dims = 1:30, resolution = 0.1; For fibroblasts, dims = 1:20, resolution = 0.1; For myeloid cells, dims = 1:20, resolution = 0.1; For T cells, dims = 1:20, resolution = 0.1. We used the CellMarker database to annotate the types of cell subgroups according to the highly expressed genes of cell subgroups^21^. Clinical information for all samples is displayed in Table 1.

**Table 1:** Clinical information on the samples.

Patient_ID	Gender	Age	Tissue_type	Subsite	HPV_infection
P15	F	56	Primary cancer	Oral cavity	HPV-
P15	F	56	Leukoplakia	Oral cavity	HPV-
P16	M	74	Leukoplakia	Oral cavity	HPV-
P17	M	49	Leukoplakia	Oral cavity	HPV-
P21	M	69	Primary cancer	Oral cavity	HPV-
P26	M	57	Primary cancer	Oral cavity	HPV-
P30	M	54	Primary cancer	Oral cavity	HPV-
P31	M	66	Primary cancer	Oral cavity	HPV-
P33	F	65	Leukoplakia	Oral cavity	HPV-
P38	M	73	Primary cancer	Hypopharynx	HPV-
P4	F	58	Primary cancer	Oral cavity	HPV-
P51	M	53	Primary cancer	Oral cavity	HPV-
P6	M	58	Primary cancer	Oral cavity	HPV-
P60	F	56	Primary cancer	Oral cavity	HPV-
P7	M	53	Primary cancer	Oral cavity	HPV-
P8	M	77	Primary cancer	Oropharynx	HPV-
P9	F	64	Primary cancer	Oropharynx	HPV-

### Functional enrichment of gene sets

In order to explore the genes involved in biological function, we upload interested gene set to DAVID database (https://david.ncifcrf.gov/), focus on enrichment to in the process (*P* < 0.05).

### Construction of single cell pseudotime trajectories

We used monocle2 to read the count data of the expression matrix, combined the phenotype information of the cells, constructed the cds object with the newCellDataSet function, and then filtered out the genes expressed in less than 10 cells^22^. We then used differentialGeneTest function (fullModelFormulaStr = ” ~ TissueType”) to calculate differential expression of genes between the white and HNSCC groups. In the case of epithelial cells, qval < 0.01. For fibroblasts, qval < 1e-08. For myeloid cells, qval < 0.01. For NK/T cells, qval < 1e-08. Next, the reduceDimension function (max_components = 2, method = ”DDRTree”) is used to reduce the dimension, and the orderCells function is used to order the cells and complete the trajectory construction^23^. Here, we set more branches of cells in the leukoplakia group as the starting point of the trajectory.

### Branch difference representation analysis of pseudotime trajectories

We used the BEAM function to compute the differentially expressed genes between the two branches, starting from the fork point, for all cell types, qval < 0.01^24^. Then plot genes branched heatmap was used to draw the heatmap of branching differentially expressed genes using pheatmap R package (version 3.18, https://bioconductor.org/packages/release/bioc/html/heatmaps.html). Finally, the plot genes branched pseudotime function is used to show the expression of the genes of interest in both branches. Cell fate 1 corresponds to the state with smaller id while cell fate 2 corresponds to state with bigger id.

### Enrichment and survival analysis of cell subgroups in TCGA-HNSCC samples

In order to explore the correlation between each cell subgroup and survival time in TCGA-HNSC samples, we used the single-sample gene set enrichment analysis (ssGSEA) method of GSVA package to calculate the enrichment score of each cell subgroup in TCGA-HNSCC samples according to the high expression genes of each cell subgroup^25^. According to the median, all patients were divided into high ssGSEA group and low ssGSEA group, and log-rank test was used to compare the difference in survival time between the two groups.

### Statistical analysis

All data processing is done in R (version 3.6.0). Our specific methods and the functions used are described in the above section. Sangerbox (http://sangerbox.com/home.html) also offered auxiliary analysis in this paper.

## Results

### Single cell landscape of leukoplakia and HNSCC

**Figure 1:**
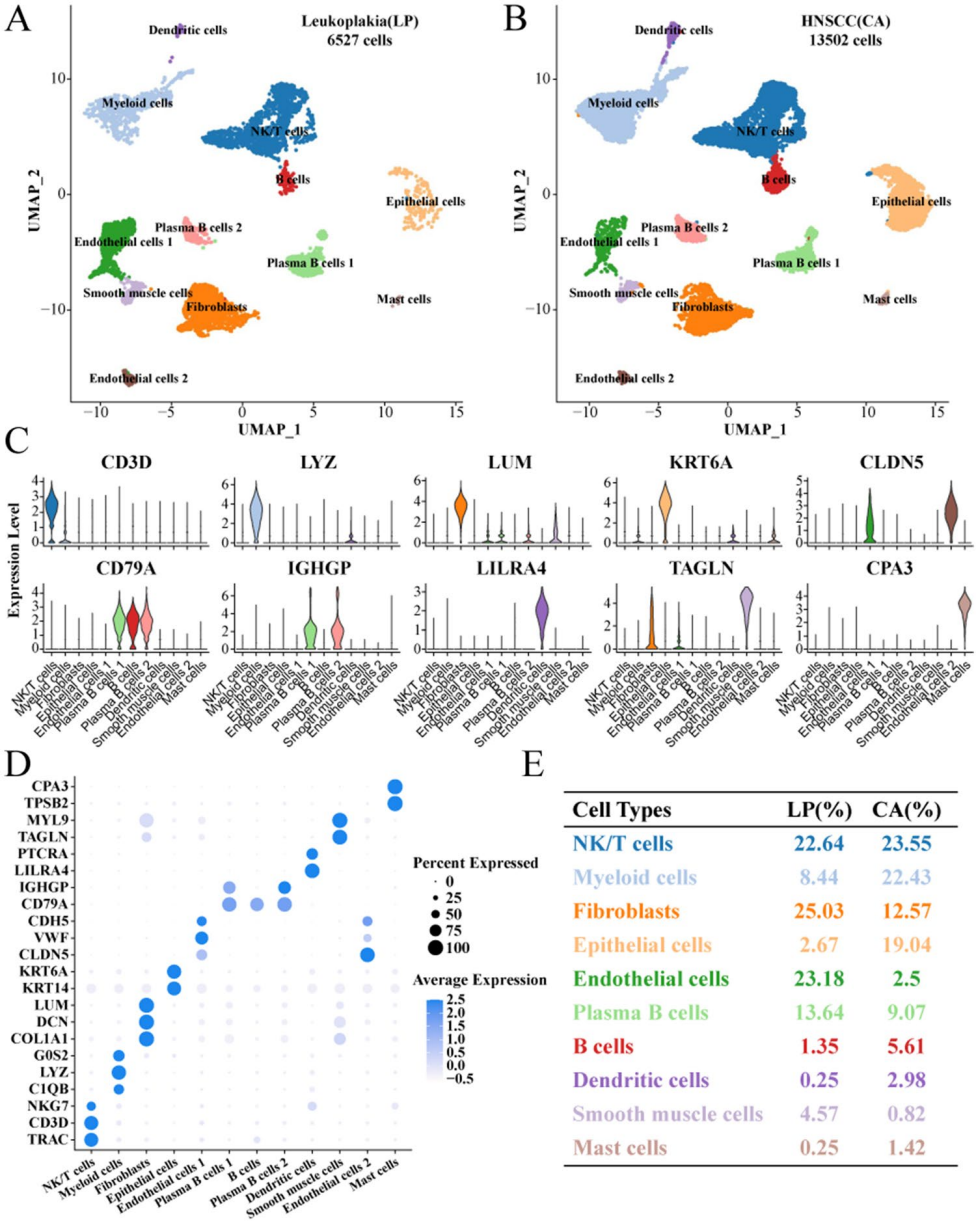
Single-cell map of leukoplakia and HNSCC. **A** Annotated UMAP of leukoplakia cell subgroups; **B** Annotated UMAP of HNSCC cell subgroups; **C** Violin map of marker gene expression in cell subgroups; **D** Bubble map of marker gene expression in cell subgroups; **E** The proportion of each cell subgroup within the leukoplakia and HNSCC groups.

After cell filtration, standardization, dimensionality reduction, and clustering, we identified a total of 12 major cell subgroups. There were 6527 cells in leukoplakia and 13,502 cells in HNSCC (Fig. 1A,B).

Next, marker genes used to annotate each cell subroup are described (Fig. 1C,D). Interestingly, we found that HNSCC had a higher proportion of NK/T cells, myeloid cells, epithelial cells, and B cells than white plaques. In contrast, the proportions of fibroblasts, endothelial cells, plasma B cells, and smooth muscle cells were all lower (Fig. 1E). These results indicate that the proliferation of epithelial cells is enhanced along with the infiltration degree of NK/T cells and myeloid cells during the progression from leuconema to HNSCC. Next, marker genes used to annotate each cell subgroup are described (Fig. 1C,D). Interestingly, we found that HNSCC had a higher proportion of NK/T cells, myeloid cells, epithelial cells, and B cells than leukoplakia. In contrast, the proportions of fibroblasts, endothelial cells, plasma B cells, and smooth muscle cells were all lower (Fig. 1E). These results indicate that the proliferation of epithelial cells is enhanced along with the infiltration degree of NK/T cells and myeloid cells during the progression from leukoplakia to HNSCC.

### Heterogeneity and differentiation trajectories of epithelial cells

**Figure 2:**
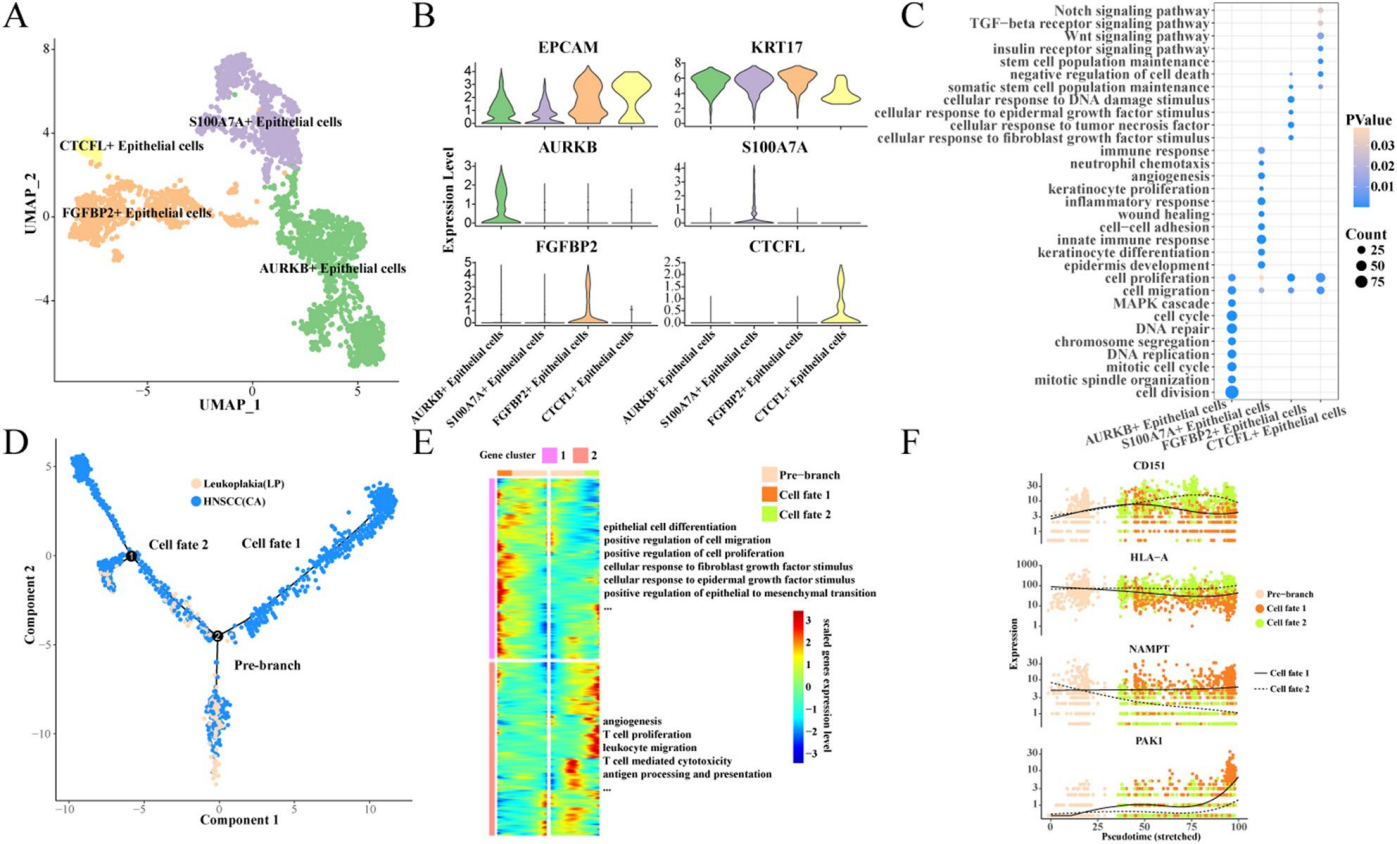
Heterogeneity and differentiation trajectories of epithelial cells. (**A**) UMAP of further subdivision of epithelial cells; (**B**) Violin map of highly expressed genes in epithelial cell subgroups; (**C**) Biological process of high-expression gene enrichment in epithelial cell subgroups; (**D**) Differentiation of epithelial cells from leukoplakia to HNSCC; (**E**) Heat map of differential gene expression and enriched biological process; (**F**) Scatter plot of the expression of branching differential genes.

Because HNSCC is closely associated with the infinite proliferation of epithelial cells, it is important to analyze the heterogeneity of epithelial cells and explore the patterns of gene expression changes from precancerous lesions to cancer. Here, we further divide epithelial cells into 4 subgroups: CTCFL + Epithelial cells, FGFBP2 + Epithelial cells, S100A7A + Epithelial cells, AURKB + Epithelial cells (Fig. 2A). We show the specific high-expression genes of each subgroup (Fig. 2B).

In order to further characterize the biological functions involved in each subgroup of epithelial cells, we functionally enriched the highly expressed genes of each subgroup, and found that AURKB + epithelial cells were mainly closely related to cell division and cell cycle. S100A7A + epithelial cells are mainly enriched in inflammation and immune response, angiogenesis, wound healing, and neutrophil chemotaxis. The high expression of FGFBP2 + gene is closely related to epidermal growth factor stimulation, fibroblast growth factor stimulation and DNA damage stimulation. CTCFL + epithelial cells are enriched in NOTCH, WNT, TGF-β, and insulin receptor signaling pathways. In particular, both FGFBP2 + epithelial cells and CTCFL + epithelial cells are associated with the maintenance of stem cell populations and negative regulation of cell death (Fig. 2C). Further, we found that high enrichment scores of AURKB + epithelial cells were associated with worse prognosis of TCGA-HNSCC, while enrichment scores of other epithelial cell subgroups were not statistically correlated with the prognosis of TCGA-HNSCC (Fig. S2).

Next, we found that epithelial cells have two main cell fates during the evolution from leukoplakia to HNSCC (Fig. 2D). One is the gradual enhancement of cell proliferation and migration, epidermal and fibroblast growth factor stimulation, and mesenchymal transformation of epithelial cells; the other is the gradual enhancement of angiogenesis, antigen presentation, promotion of T cell proliferation and killing (Fig. 2E). We observed the expression of four differential genes in different cell fates, and found that with the development of Pseudotime, the expression of CD151 and HLA-A in cell Fates 2 was gradually higher than that in cell fates 1, while the expression of NAMPT and PAK1 in cell fates 2 was significantly lower than that in cell fates 1 (Fig. 2F).

### Heterogeneity and differentiation trajectories of fibroblasts

As cancer-associated fibroblasts (CAFs) can secrete a variety of cytokines, growth factors and extracellular matrix proteins, they promote the proliferation, drug resistance and invasion and metastasis of tumor cells, thus affecting the prognosis of tumors. Therefore, we then characterize the heterogeneity and differentiation trajectories of fibroblasts.

**Figure 3:**
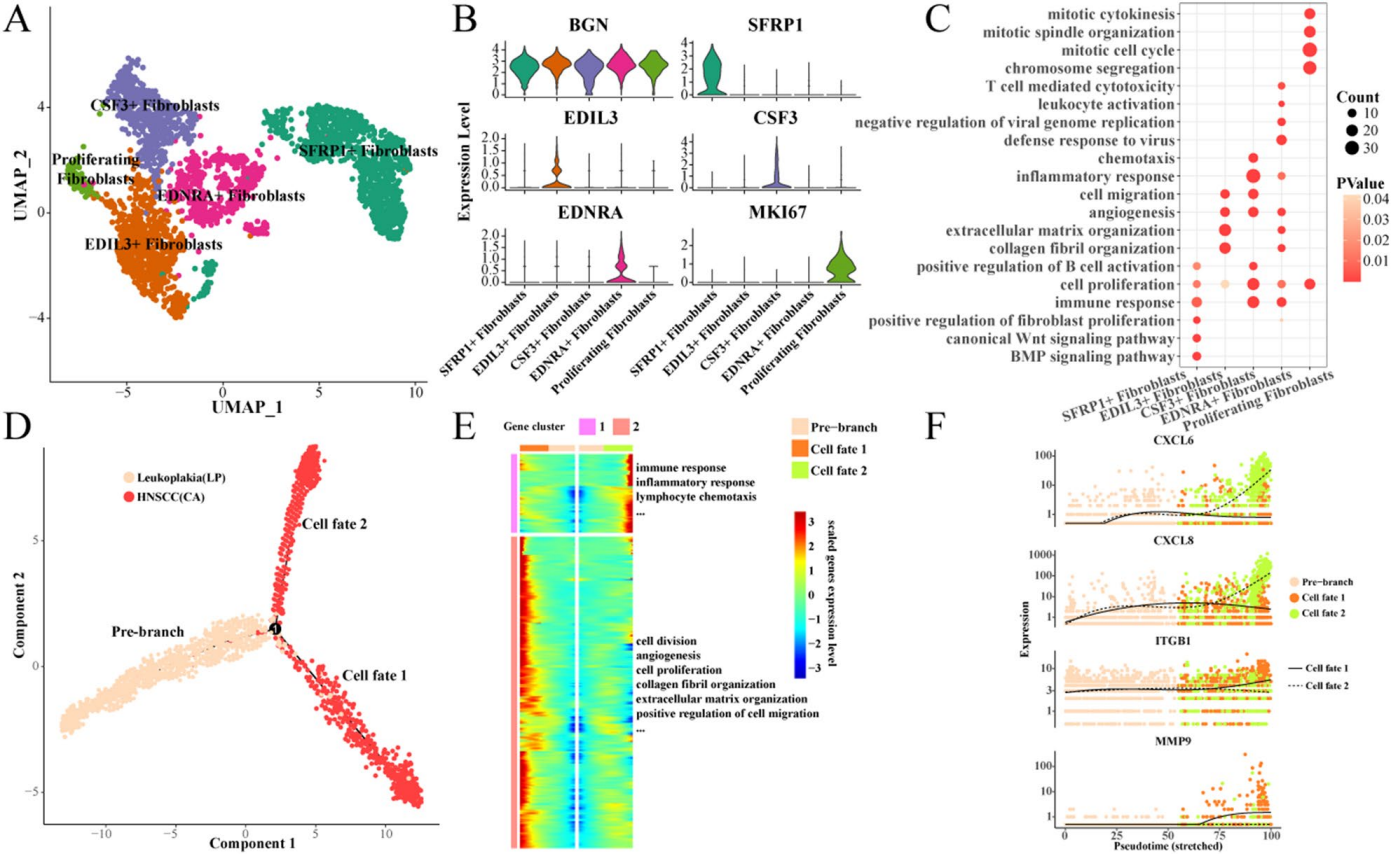
Heterogeneity and differentiation trajectories of fibroblasts. (**A**) UMAP of further subdivision of fibroblasts; (**B**) Violin map of highly expressed genes in fibroblast subgroups; (**C**) Biological process of high-expression gene enrichment in fibroblast subgroups; (**D**) Differentiation trajectory of fibroblasts from leukoplakia to HNSCC; (**E**) Heat map of differential gene expression and enriched biological process; (**F**) Scatter plot of the expression of branching differential genes.

We further divided fibroblasts into five subgroups and showed the specific high-expression genes of each subgroup (Fig. 3A,B). Similarly, we then characterize the biological functions involved in each fibroblast subgroup and find that SFRP1 + fibroblasts are associated with BMP signaling pathways and classical WNT signaling pathways. EDIL3 + fibroblasts and EDNRA + fibroblasts are related to collagen fiber composition, extracellular matrix composition and angiogenesis. CSF3 + fibroblasts are closely related to cell migration, chemotaxis and inflammation. In particular, EDNRA + fibroblasts were enriched in T cell-mediated killing, leukocyte activation, and viral resistance. Proliferating fibroblasts overexpress many genes related to cell proliferation (Fig. 3C). Further, we found that except for SFRP1 + fibroblasts, high enrichment scores of other fibroblast subgroups were associated with worse prognosis of TCGA-HNSC (Fig. S3).

Next, we found that fibroblasts have two main cell fates during the evolution from leukoplakia to HNSCC (Fig. 3D). One favors inflammation and immune responses, as well as leukocyte chemotaxis. The other tends to be cell proliferation and migration, angiogenesis, and extracellular matrix composition (Fig. 3E). We observed the expression of four differential genes in different cell fates, and found that with the development of Pseudotime, the expression of CXCL2 and CXCL8 in cell Fates 2 was gradually higher than that in cell fates 1, while the expression of ITGB1 and MMP9 in cell fates 2 was significantly lower than that in cell fates 1 (Fig. 3F).

### Heterogeneity and differentiation trajectories of myeloid cells

There are many types of myeloid cells, which play different roles in the immune response, including dendritic cells, macrophages, granulocytes, etc. Tumor-associated macrophages (TAMs) are the most abundant innate immune population in the tumor microenvironment, with heterogeneity and differentiable plasticity ranging from anti-tumor to pro-tumor.

**Figure 4:**
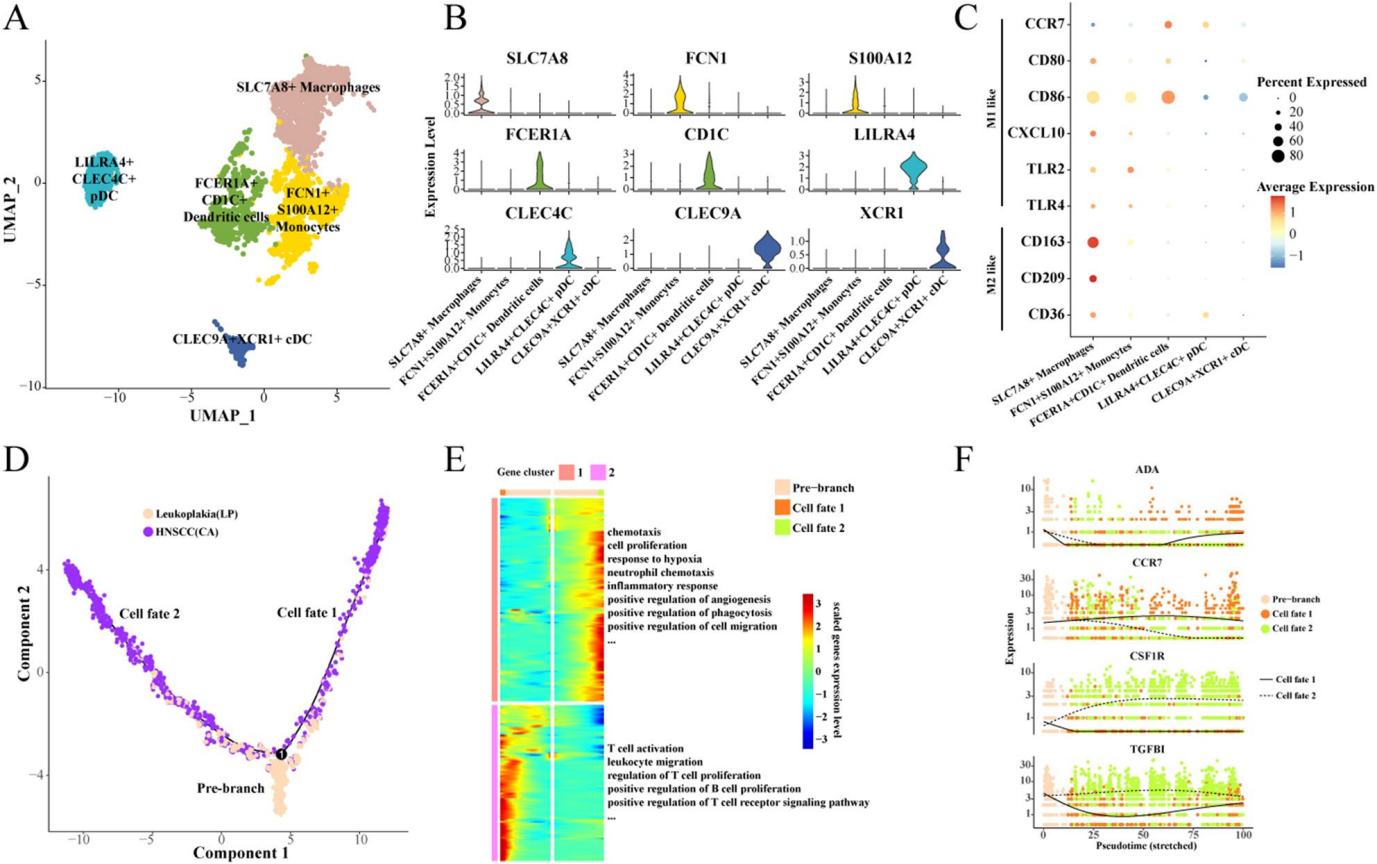
Heterogeneity and differentiation trajectories of myeloid cells. (**A**) UMAP of further subdivision of myeloid cells; (**B**) Violin map of highly expressed genes in myeloid cell subgroups; (**C**) Bubble map of M1 and M2 macrophage marker gene expression among myeloid cell subgroups; (**D**) Differentiation trajectory of myeloid cells from leukoplakia to HNSCC; (**E**) Heat map of differential gene expression and enriched biological process; (**F**) Scatter plot of the expression of branching differential genes.

Here, we subdivide myeloid cells into 5 subgroups: LILR4 + CLEC4C + pDC, SLC7A8 + macrophages, FCER1A + CD1C + dendritic cells, FCN1 + S100A12 + monocytes, CLEC9A + XCR1 + cDC (Fig. 4A). We show the highly expressed genes in each subgroup (Fig. 4B). Interestingly, we found three types of dendritic cells. Further, we found that SLC7A8 + macrophages highly expressed CD163 and CD209 and other marker genes of M2 macrophages, which played a role in promoting tumor, inhibiting immune response, inducing angiogenesis and tissue repair (Fig. 4C). High enrichment of all three types of dendritic cells was associated with better prognosis of TCGA-HNSCC, while the enrichment of SLC7A8 + macrophages and FCN1 + S100A12 + monocytes was not associated with the prognosis of TCGA-HNSCC (Fig. S4).

Next, we found that there are two main different fates of myeloid cells during the evolution from leukoplakia to HNSCC (Fig. 4D). One favors chemotaxis, cell proliferation and migration, inflammatory responses, and angiogenesis. The other favors activation of T and B cells (Fig. 4E). We observed the expression of four differential genes in different cell fates, and found that with the development of Pseudotime, the expression of CSF1R and TGFBI in cell Fates 2 was gradually higher than that in cell Fates 1, while the expression of ADA and CCR7 in cell fates 2 was significantly lower than that in cell fates 1 (Fig. 4F).

Finally, we found that the myeloid cells subgroup in the HNSCC group highly expressed the marker genes of M2 macrophages such as CD163 and CD209, indicating that the tumor promoting effect of myeloid cells in the HNSCC group was stronger than that of leukoplakia (Fig. S5).

### Heterogeneity and differentiation trajectories of NK/T cells

**Figure 5:**
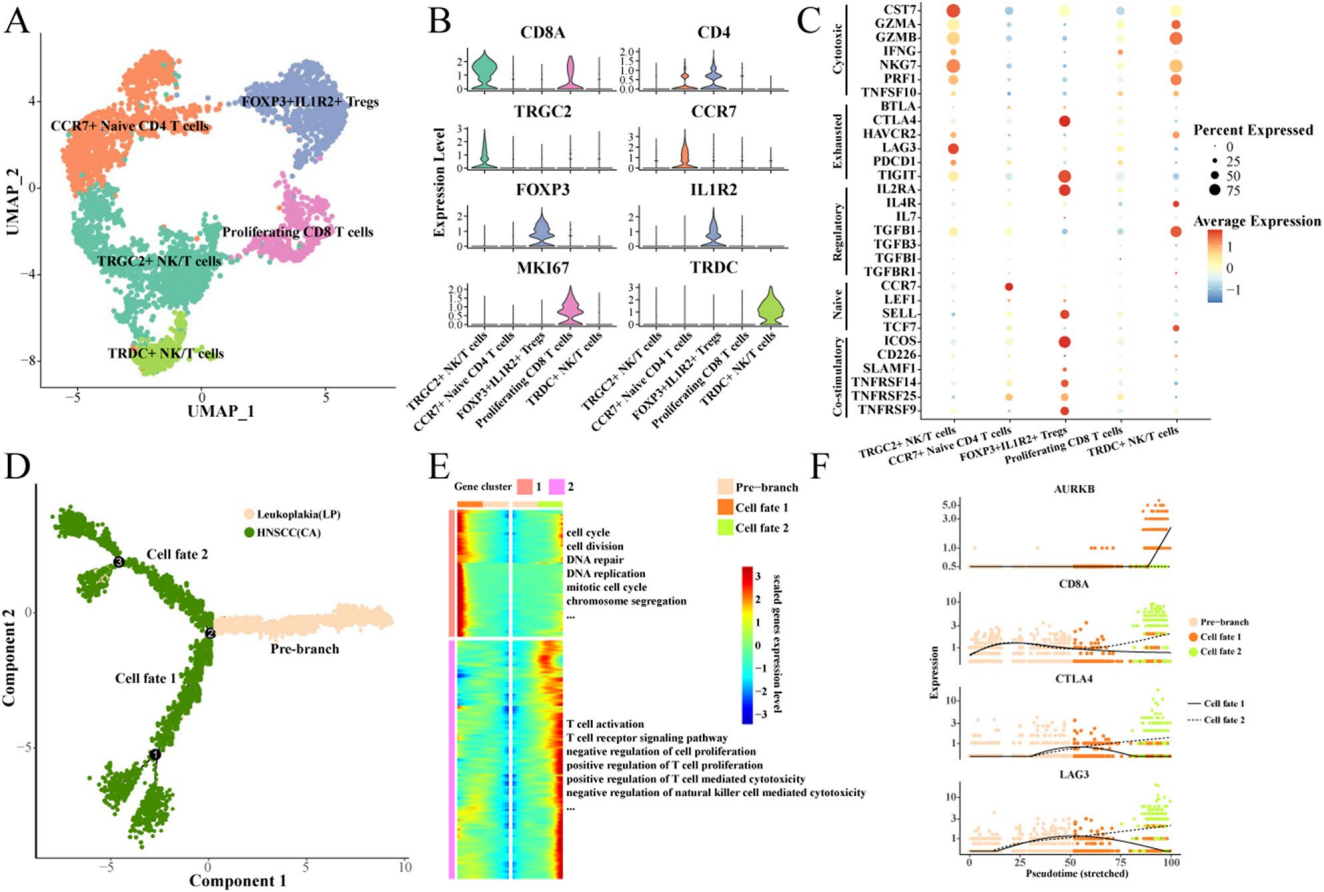
Heterogeneity and differentiation trajectories of NK/T cells. (**A**) UMAP for further subdivision of NK/T cells; (**B**) Violin map of NK/T cell subgroups with high expression genes; (**C**) Bubble map of the expression of marker genes such as kill, depletion and initial among NK/T cell subgroups; (**D**) Differentiation trajectory of NK/T cells from leukoplakia to HNSCC; (**E**) Heat map of differential gene expression and enriched biological process; (**F**) Scatter plot of the expression of branching differential genes.

In order to characterize the functional status of NK/T cell subgroups in HNSCC, we first further subdivided NK/T cells into 5 subgroups: CCR7 + Naive CD4 T cells, FOXP3 + IL1R2 + Tregs, TRGC2 + NK/T cells, Proliferating CD8 T cells, and TRDC + NK/T cells (Fig. 5A). Our analysis looked at the specific high-expression genes in each subgroup (Fig. 5B).

We found that both TRGC2 + NK/T cells and TRDC + NK/T cells highly expressed killer-related marker genes, but TRGC2 + NK/T cells highly expressed LAG3, and TRDC + NK/T cells highly expressed TGFB1. These results indicate that these two cell subgroups are also immunosuppressed. In particular, FOXP3 + IL1R2 + Tregs highly expressed CTLA4, TIGIT, and IL2RA, indicating that this subgroup was already in a state of exhaustion (Fig. 5C). Interestingly, we found that high enrichment of TRGC2 + NK/T cells, TRDC + NK/T cells, and CCR7 + Naive T cells was associated with a better prognosis for TCGA-HNSCC. However, FOXP3 + IL1R2 + Tregs and Proliferating CD8 T cells were not associated with the prognosis of TCGA-HNSC (Fig. S6).

Next, we found that NK/T cells have two main cell fates during the evolution from leukoplakia to HNSCC (Fig. 5D). One is a gradual increase in the expression of genes associated with cell cycle and division. The other is a gradual increase in the expression of genes associated with T cell self-activation (Fig. 5E). We observed the expression of four differential genes in different cell fates, and found that with the development of Pseudotime, the expression of CD8A, CTLA4, and LAG3 in cell Fates 2 was gradually higher than that in cell fates 1, while the expression of AURKB in cell fates 2 was significantly lower than that in cell fates 1 (Fig. 5F).

Finally, we found that NK/T cell subgroups in the HNSCC group highly expressed marker genes of depleted T cells such as CTLA4, LAG3, TIGIT, and HAVCR2, indicating that the inhibition degree of NK/T cells in the HNSCC group was stronger than that of leukoplakia (Fig. S7).

## Discussion

HNSCC includes various cancers that originate from the epithelial cells of the upper aerodigestive tract, leading to a substantial global health burden because of the high rates of mortality, morbidity, and tendency for recurrence associated with the disease. The prognosis for HNSCC in cases of recurrence or metastasis is particularly grim, and the response rates to immunotherapy are typically inadequate^26^. However, recent studies of the immune microenvironment of HNSCC have revealed a more complex picture, some of which have found variable degrees of T cell and NK cell penetration in HNSCC. In their study, Zhang et al. found an immunologically active subpopulation of HNSCC patients that exhibited higher levels of CD8 + T cell and NK cell activity^27^. There are studies confirming that the expression of NK cell markers is significantly increased in HNSCC samples compared to healthy controls^28,29^. Furthermore, Choi et al. focused on the progressive progression of HNSCC from normal tissue to precancerous white spots, primary HNSCC, and metastatic tumors. They emphasized the role of LAIR2 expression in regulatory T cells in promoting tumor growth^30^. In this study, we used single-cell RNA sequencing to explore the evolution from leukoplakia to HNSCC identified several prognostic cell subpopulations associated with the progression from leukoplakia to HNSCC and discussed the molecular mechanisms behind this transition. In particular, we focused on differential gene expression in different cell types and constructed differentiation trajectories to understand progression from leukoplakia to HNSCC and ecosystem changes.

In this study, we found that AURKB + epithelial cell enrichment gene is mainly related to cell division and cell cycle. During the evolution of leukoplakia to HNSCC, epithelial cells have two cell fates. It was found that with the development of Pseudotime, the expression of CD151 and HLA-A in cell fate 2 was gradually higher than that in cell fate 1, while the expression of NAMPT and PAK1 in cell date 2 was significantly lower than that in cell fate 1. NAD is closely related to many important life processes such as cell metabolism, aging, apoptosis, DNA damage and repair. Nicotinamide phosphoribosyltransferase (NAMPT) is the speed limit of NAD biosynthesis enzyme, several studies have found that NAMPT expressed in all kinds of tumor tissue, It may participate in signal transduction related to cell carcinogenesis and affect cell metabolism through NAD pathway, which may lead to the occurrence and progression of cancer^31^. The p21-activated kinases 1(PAK1) has been shown to be a downstream node of multiple oncogenic signaling pathways that promote tumor changes.Venu et al. demonstrated that PAK1 and its activation state have the ability to progress toward a more aggressive phenotype in gliomas^32^. CD151, a member of the tetraspanin family, has been shown to participate in the process of tumor spread, invasion, and help tumor angiogenesis^33^. HLA-A, a product of the human leucocyte antige I gene, may regulate the recognition of tumors by homologous T cells and NK cells^34,35^. Anand G Menon et al. found that downregulation of HLA-A expression may improve the activity of NK cells and produce a better prognosis^36^.

Fibroblasts also have two cellular fates in the progression of HNSCC. It was found that with the development of Pseudotime, the expression of CXCL2 and CXCL8 in cell fate 2 was gradually higher than that in cell fate 1, while the expression of ITGB1 and MMP9 in cell fate 2 was significantly lower than that in cell fate 1. CXCL2 and CXCL8 are two types of chemokine (C-X-C motif) ligand (CXCL), which are generally produced by monocytes and can recruit neutrophils to accumulate in the tumor environment, causing immunosuppression and promoting cancer production and progression^37,38^. Integrinβ1 (ITGB1) is an extracellular matrix receptor that is often involved in angiogenesis, cell cycle regulation, and cancer invasion. Wang et al. found that Ropivacaine was able to pass through ITGB1 in order to inhibit the proliferation and migration of colorectal cancer cells^39^. Fei et al. found that ITGB1 was positively correlated with most indicators of cell proliferation in a gene co-expression study. This suggests that ITGB1 has an important role in controlling cancer metastasis and proliferation^40^. Matrix metalloproteinase-9 (MMP-9) is a Matrix metalloproteinase that helps degrade parts of the extracellular matrix and has also been found to be involved in processes such as inflammation, cancer invasion and metastasis^41^. Luying He et al. also found that MMP9 can also regulate the activity and distribution of a variety of immune cells to affect the function of the immune system, leading to a poor prognosis of cancer^42^.

In isolating subgroups of myeloid cells, we found three different types of dendritic cells. Dendritic cells are antigen-presenting cells that are essential for the activation of CD4 + T cells and are important players in adaptive immune responses^43,44^. Dendritic cells are highly heterogeneous and are generally divided into conventional dendritic cells (cDC) and plasmacytoid dendritic cells (pDC)^45^. Dendritic cells can present tumor-associated antigens to naive T cells, producing specific T cells with anti-tumor functions that help inhibit the progression of cancer^46^. The differential genes we studied, ADA and CCR7, are also genes involved in activating T cells. CCR7 chemokine receptors mediate dendritic cell migration and help activate immune processes^47^. Research on the CCR7 axis has been found to help develop new therapeutic targets and is expected to improve immunotherapy strategies for cancer^48^.

NK/T cell subgroups in HNSCC group highly expressed marker genes of depleted T cells such as CTLA4, LAG3, TIGIT, HAVCR2, etc., indicating that tumor tissue was more sensitive to NK/T cells. Cytotoxic T lymphocyte antigen 4 (CTLA4) is a common target of T cells with high expression in tumor patients, which can bind to B7 ligand on tumor cells to inhibit T cell activity^49^. Using an immunoreactive transgenic mouse model with spontaneous HNSCC, Yu et al. found that targeting CTLA4 in the tumor microenvironment reduced myeloid-derived suppressor cells and macrophage levels and promoted T cell activation. Lymphocyte Activation Gene 3 (LAG3) is a key gene that regulates T cells, and its overexpression may lead to T cell depletion and ultimately poor tumor prognosis^50^. To date, immunotherapy targeting LAG3 has been demonstrated as an important anticancer agent in clinical trials for multiple cancer types^51^. Zhou et al. showed that the advantage of LAG3 blockade compared to PD-L1 blockade was more pronounced in tumor antigen-specific stimulation and concluded that LAG3 can be a promising target for immunotherapy against colorectal cancer^52^. In our study, it was indeed observed that in cell fate 2, higher levels of CTLA4 and LAG3 were expressed compared to cell fate 1. This finding instructs that cells in cell fate 2 may have a stronger immunosuppressive profile, which is associated with a T-cell depleted state.

There are some limitations in this study. In this study, the heterogeneity of epithelial cells, fibroblasts, myeloid cells, and NK/T cells was analyzed in order to find the biological processes involved in each cell subgroup. However, whether there is a link between different cell subgroups is unclear, and the specific biological processes and molecular mechanisms of evolution need to be further studied. This study will provide a key reference for the molecular mechanism exploration of leukoplakia progression to HNSCC and the immune landscape research.

## Conclusion

We identified a variety of cell subgroups associated with HNSCC prognosis through scRNA-seq. The differentiation trajectory of each cell subgroup from leukoplakia to HNSCC was constructed successively, revealing the ecosystem progress in the evolution of HNSCC. We hope that our study can provide new ideas for exploring the molecular mechanism of HNSCC, help develop new immunotherapy drugs, and improve clinical treatment strategies.

### Supplementary Information

Supplementary Legends.

Supplementary Figure S1.

Supplementary Figure S2.

Supplementary Figure S3.

Supplementary Figure S4.

Supplementary Figure S5.

Supplementary Figure S6.

Supplementary Figure S7.

## Data Availability

The dataset used in this study is available in GSE181919 (https://www.ncbi.nlm.nih.gov/geo/query/acc.cgi?acc=GSE181919).

## Abbreviations

HNSCC: Head and neck squamous cell carcinoma
GEO: Gene expression omnibus
scRNA-seq: Single-cell RNA sequencing
TAMs: Tumor-associated macrophages
PD-1: Programmed death 1
CTLA 4: Cytotoxic T lymphocyte antigen 4
ssGSEA: Single-sample gene set enrichment analysis
CAFs: Cancer-associated fibroblasts
NAMPT: Nicotinamide phosphoribosyltransferase
PAK1: P21-activated kinases 1
CXCL: Chemokine (C-X-C motif) ligand
MMP9: Matrix metalloproteinase-9
ITGB1: Integrinβ1
cDC: Conventional dendritic cells
pDC: Plasmacytoid dendritic cells
LAG3: Lymphocyte activation gene 3
