# Supplementary material for: Single-cell RNA sequencing explores the evolution of the ecosystem from leukoplakia to head and neck squamous cell carcinoma
Source: Sci Rep. 2024 Apr 6;14:8097. doi: 10.1038/s41598-024-58978-9 (PMC10998855; doi:10.1038/s41598-024-58978-9)
Supplement: Supplementary file 1 — Supplementary Legends. [file 41598_2024_58978_MOESM1_ESM.docx]

**Description of supplementary figure legends**

**Figure S1** Filtering, standardization, dimensionality reduction and clustering of scRNA-seq date

A: After the batch effect is removed with the harmony package, the cells in different samples are evenly distributed together; B: The distribution of the number of genes detected in the cells of each sample (between 200 and 8000); C: The distribution of the proportion of mitochondrial genes detected within the cells of each sample (<10%)

**Figure S2** Survival curve of enrichment score for each epithelial cell subgroup in TCGA-HNSCC

**Figure S3** Survival curve of enrichment score of each fibroblast subgroup in TCGA-HNSCC

**Figure S4** Survival curve of enrichment score of each myeloid cell subgroup in TCGA-HNSCC

**Figure S5** Expression of marker gene in M2 macrophage between leukoplakia and HNSCC

**Figure S6** Survival curve of enrichment score of each NK/T cell subgroup in TCGA-HNSCC

**Figure S7** Expression of depletion T cell marker gene between leukoplakia and head and HNSCC
