# Supplementary figures and images for: Single-cell RNA sequencing explores the evolution of the ecosystem from leukoplakia to head and neck squamous cell carcinoma
Source: Sci Rep. 2024 Apr 6;14:8097. doi: 10.1038/s41598-024-58978-9 (PMC10998855; doi:10.1038/s41598-024-58978-9)

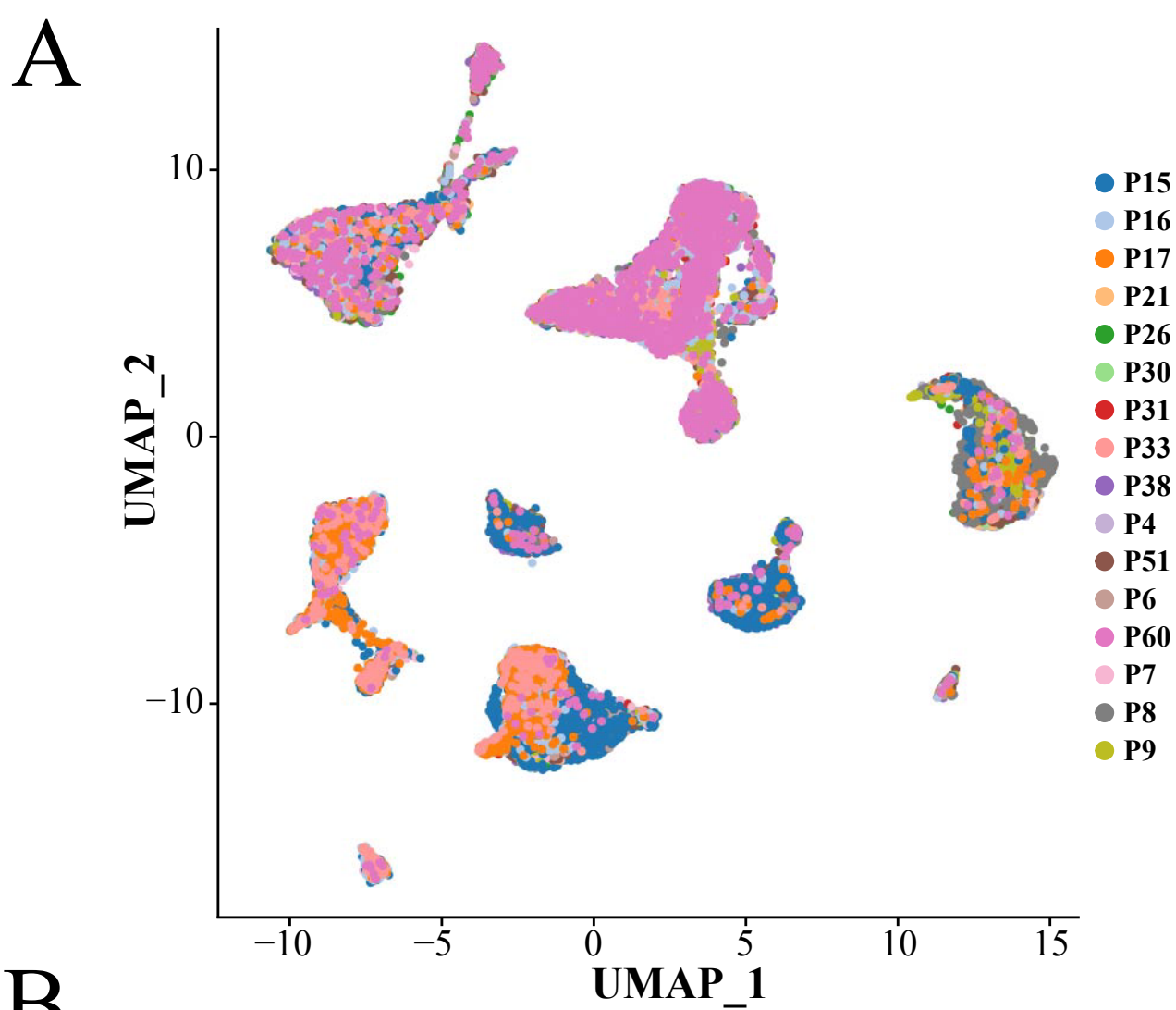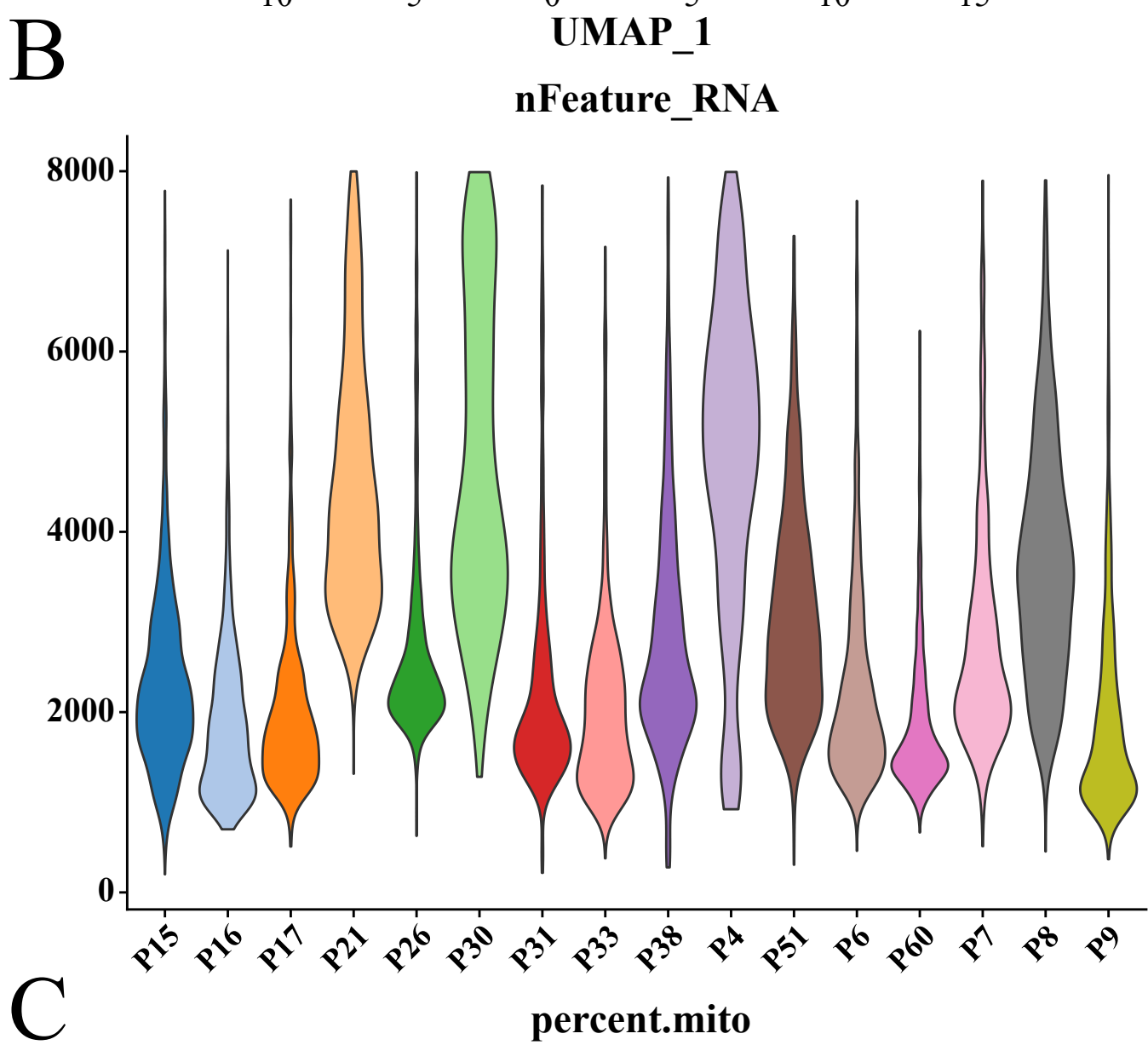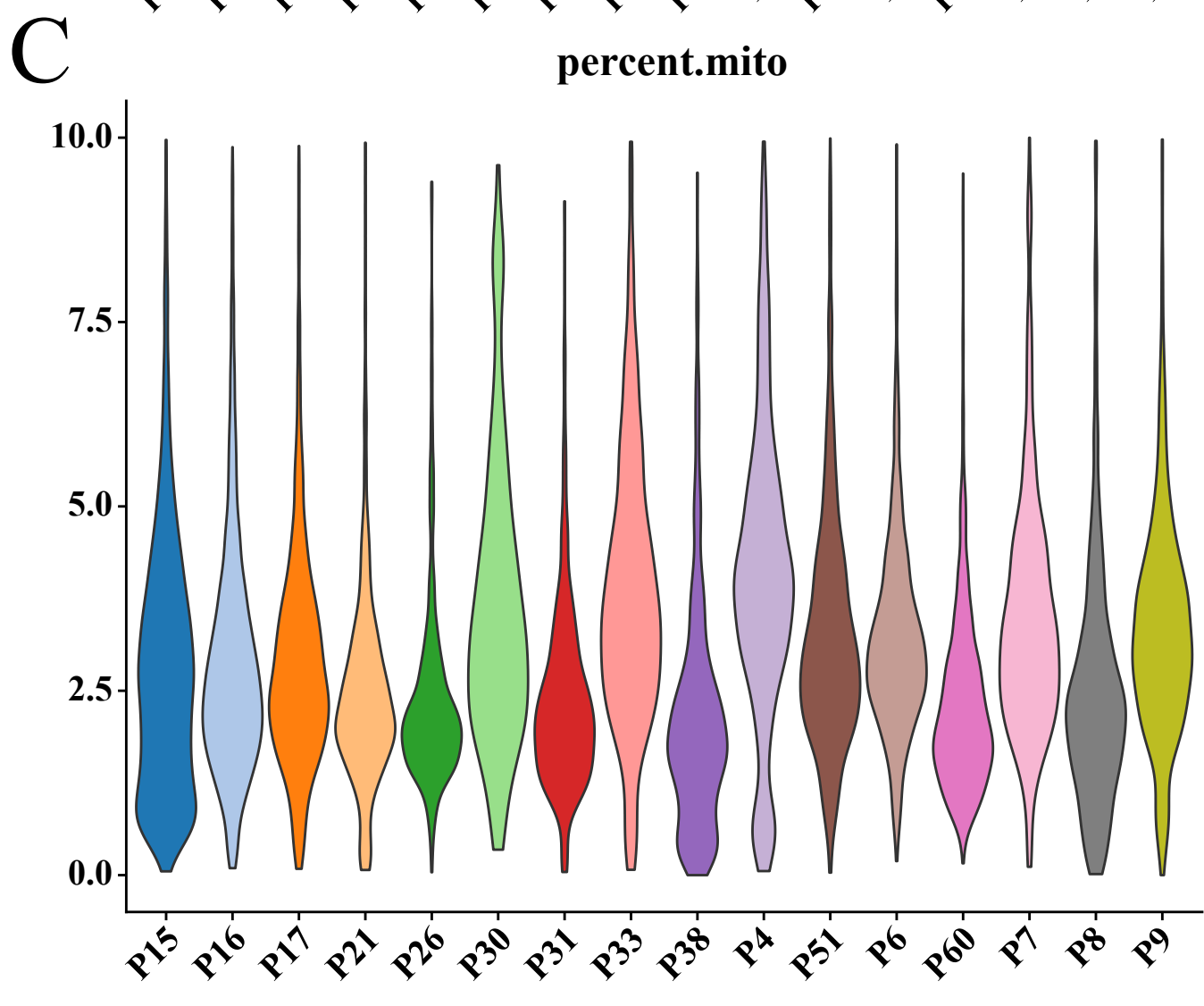

Supplement: Supplementary file 2 — Supplementary Figure S1. [file 41598_2024_58978_MOESM2_ESM.pdf]

## CD163

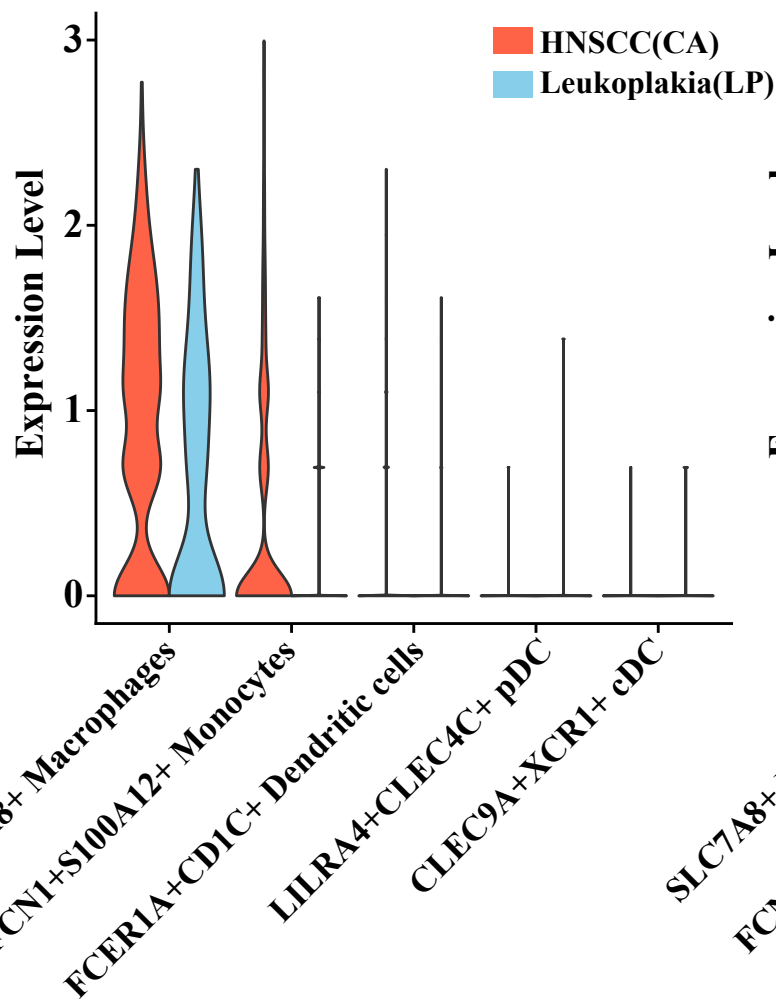

## CD209

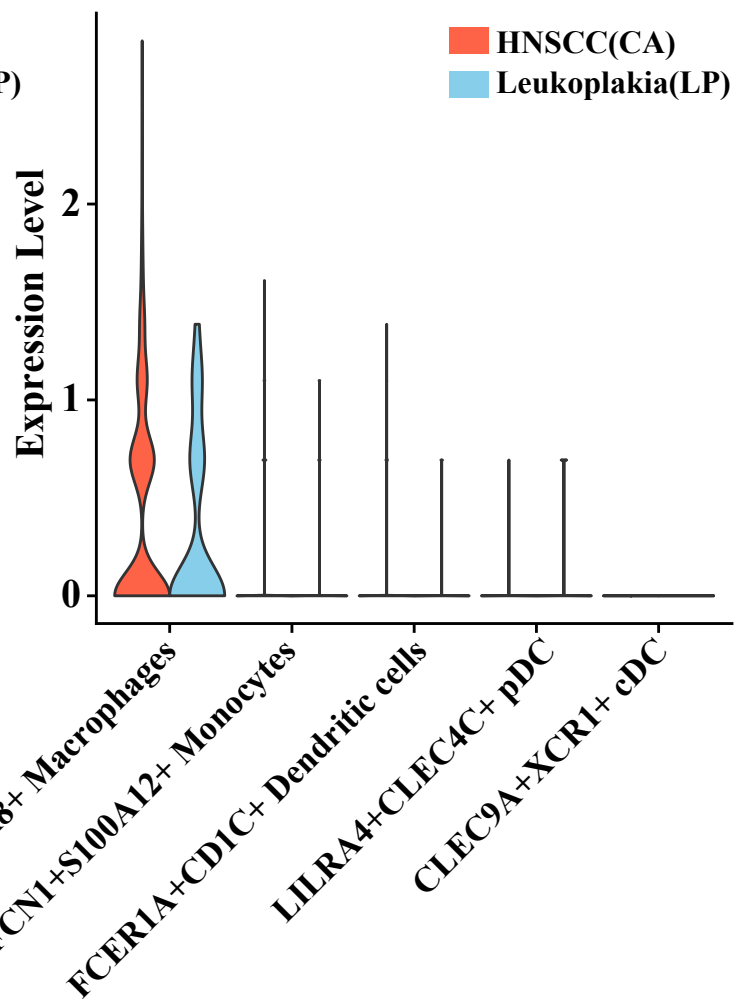

Supplement: Supplementary file 6 — Supplementary Figure S5. [file 41598_2024_58978_MOESM6_ESM.pdf]

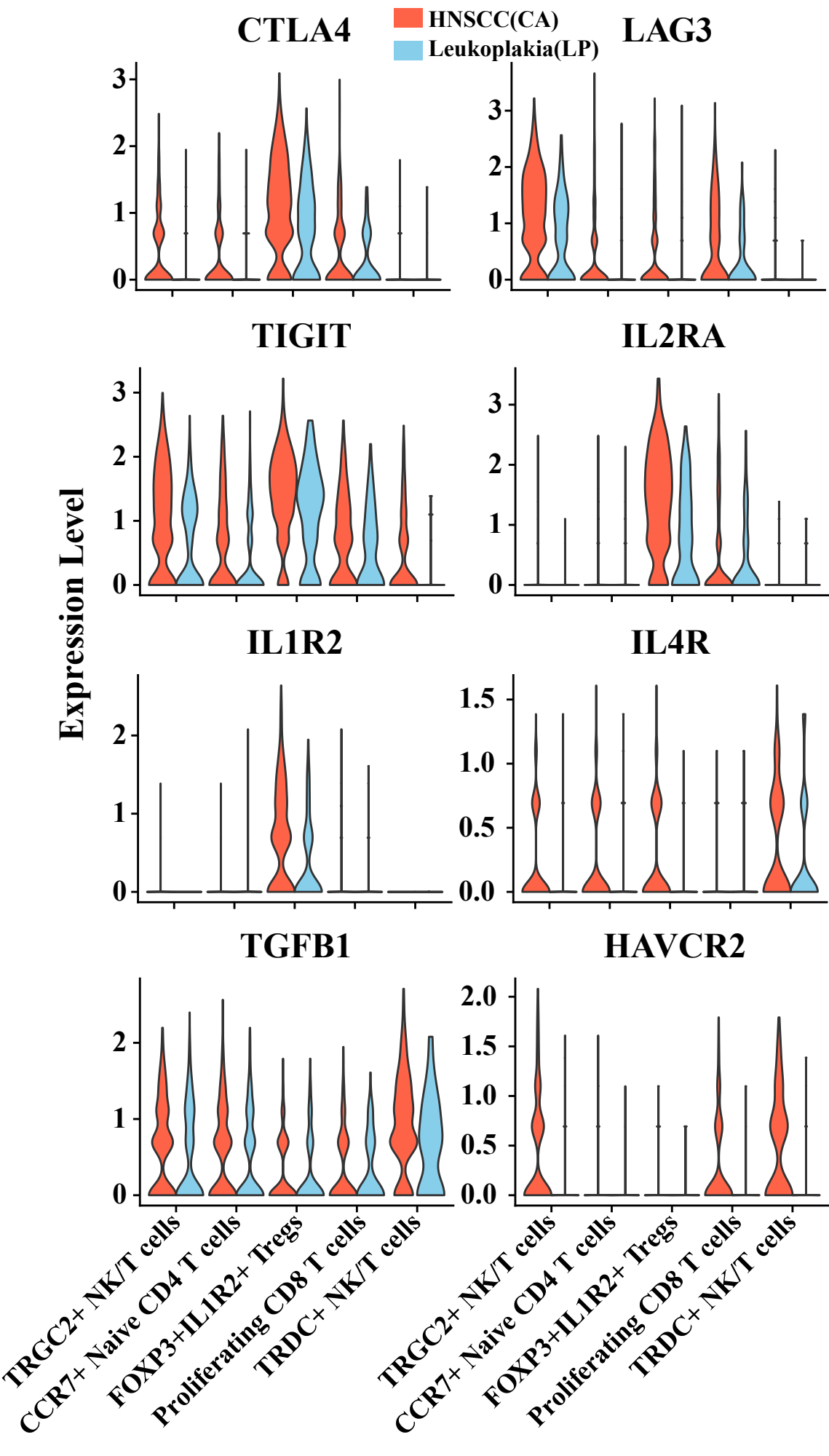

Supplement: Supplementary file 8 — Supplementary Figure S7. [file 41598_2024_58978_MOESM8_ESM.pdf]
