# Supplementary material for: Single-cell RNA sequencing explores the evolution of the ecosystem from leukoplakia to head and neck squamous cell carcinoma
Source: Sci Rep. 2024 Apr 6;14:8097. doi: 10.1038/s41598-024-58978-9 (PMC10998855; doi:10.1038/s41598-024-58978-9)
Supplement: Supplementary file 3 — Supplementary Figure S2. [file 41598_2024_58978_MOESM3_ESM.pdf]

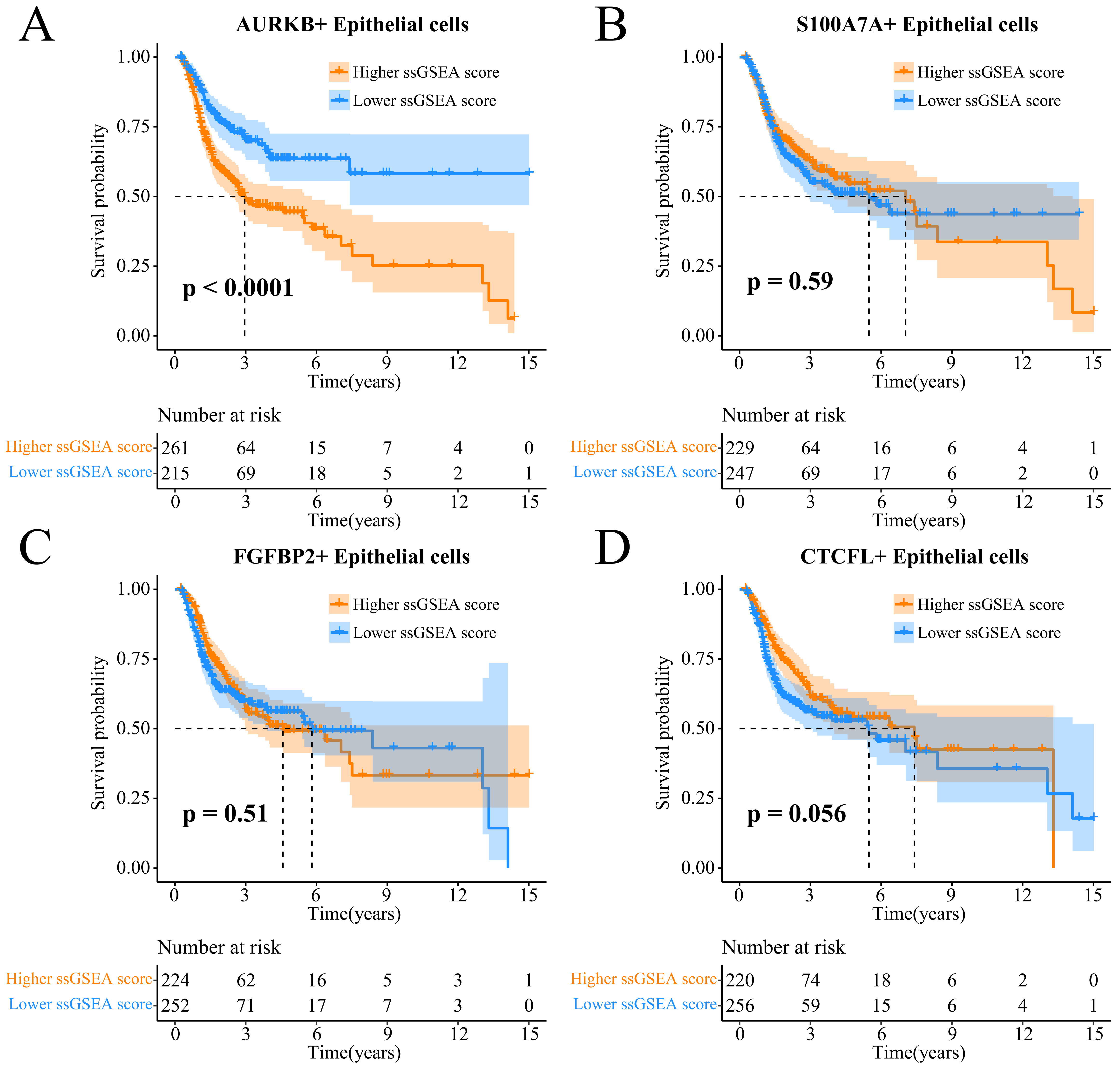
A
AURKB+ Epithelial cells
Higher ssGSEA score
Lower ssGSEA score
Survival probability
p < 0.0001
Time(years)
Number at risk
Higher ssGSEA score 261 64 15 7 4 0
Lower ssGSEA score 215 69 18 5 2 1
B
S100A7A+ Epithelial cells
Higher ssGSEA score
Lower ssGSEA score
Survival probability
p = 0.59
Time(years)
Number at risk
Higher ssGSEA score 229 64 16 6 4 1
Lower ssGSEA score 247 69 17 6 2 0
C
FGFBP2+ Epithelial cells
Higher ssGSEA score
Lower ssGSEA score
Survival probability
p = 0.51
Time(years)
Number at risk
Higher ssGSEA score 224 62 16 5 3 1
Lower ssGSEA score 252 71 17 7 3 0
D
CTCFL+ Epithelial cells
Higher ssGSEA score
Lower ssGSEA score
Survival probability
p = 0.056
Time(years)
Number at risk
Higher ssGSEA score 220 74 18 6 2 0
Lower ssGSEA score 256 59 15 6 4 1
