# Supplementary material for: Single-cell RNA sequencing explores the evolution of the ecosystem from leukoplakia to head and neck squamous cell carcinoma
Source: Sci Rep. 2024 Apr 6;14:8097. doi: 10.1038/s41598-024-58978-9 (PMC10998855; doi:10.1038/s41598-024-58978-9)
Supplement: Supplementary file 4 — Supplementary Figure S3. [file 41598_2024_58978_MOESM4_ESM.pdf]

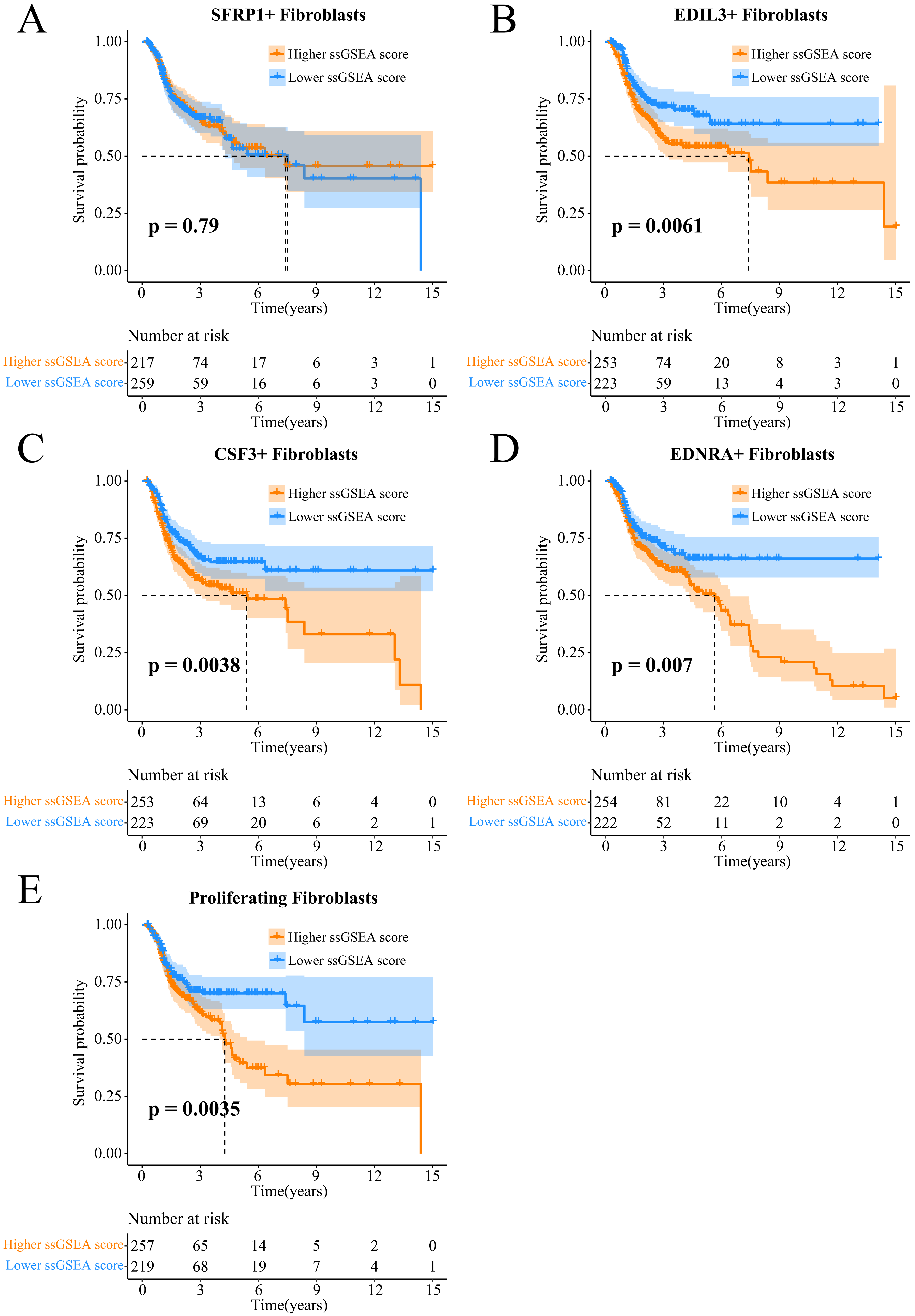
A
SFRP1+ Fibroblasts
Higher ssGSEA score
Lower ssGSEA score
Survival probability
1.00
0.75
0.50
0.25
0.00
p = 0.79
0 3 6 9 12 15
Time(years)
Number at risk
Higher ssGSEA score 217 74 17 6 3 1
Lower ssGSEA score 259 59 16 6 3 0
0 3 6 9 12 15
Time(years)
B
EDIL3+ Fibroblasts
Higher ssGSEA score
Lower ssGSEA score
Survival probability
1.00
0.75
0.50
0.25
0.00
p = 0.0061
0 3 6 9 12 15
Time(years)
Number at risk
Higher ssGSEA score 253 74 20 8 3 1
Lower ssGSEA score 223 59 13 4 3 0
0 3 6 9 12 15
Time(years)
C
CSF3+ Fibroblasts
Higher ssGSEA score
Lower ssGSEA score
Survival probability
1.00
0.75
0.50
0.25
0.00
p = 0.0038
0 3 6 9 12 15
Time(years)
Number at risk
Higher ssGSEA score 253 64 13 6 4 0
Lower ssGSEA score 223 69 20 6 2 1
0 3 6 9 12 15
Time(years)
D
EDNRA+ Fibroblasts
Higher ssGSEA score
Lower ssGSEA score
Survival probability
1.00
0.75
0.50
0.25
0.00
p = 0.007
0 3 6 9 12 15
Time(years)
Number at risk
Higher ssGSEA score 254 81 22 10 4 1
Lower ssGSEA score 222 52 11 2 2 0
0 3 6 9 12 15
Time(years)
E
Proliferating Fibroblasts
Higher ssGSEA score
Lower ssGSEA score
Survival probability
1.00
0.75
0.50
0.25
0.00
p = 0.0035
0 3 6 9 12 15
Time(years)
Number at risk
Higher ssGSEA score 257 65 14 5 2 0
Lower ssGSEA score 219 68 19 7 4 1
0 3 6 9 12 15
Time(years)
