# Supplementary material for: Single-cell RNA sequencing explores the evolution of the ecosystem from leukoplakia to head and neck squamous cell carcinoma
Source: Sci Rep. 2024 Apr 6;14:8097. doi: 10.1038/s41598-024-58978-9 (PMC10998855; doi:10.1038/s41598-024-58978-9)
Supplement: Supplementary file 5 — Supplementary Figure S4. [file 41598_2024_58978_MOESM5_ESM.pdf]

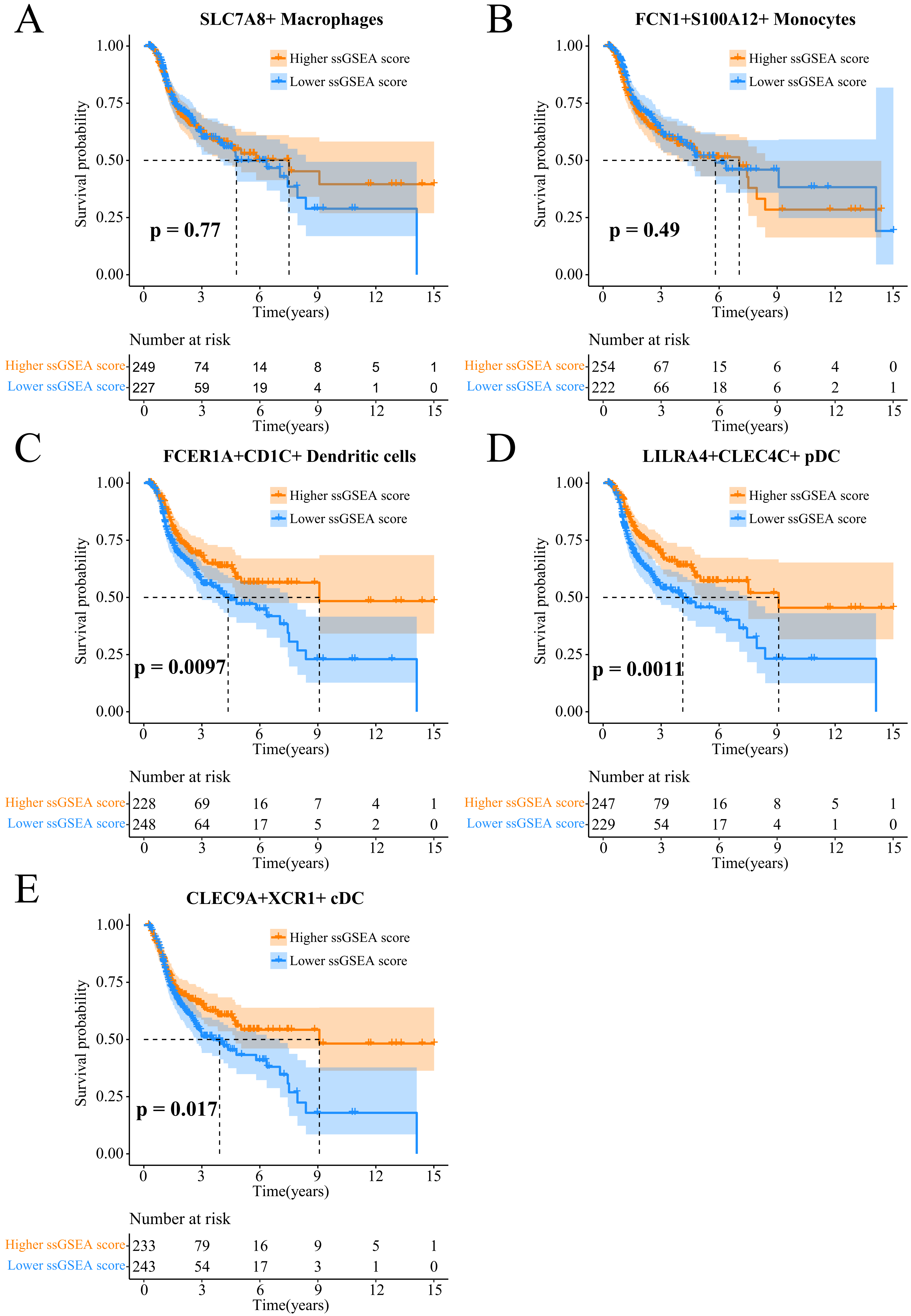
A
SLC7A8+ Macrophages
Higher ssGSEA score
Lower ssGSEA score
Survival probability
p = 0.77
Time(years)
Number at risk
Higher ssGSEA score 249 74 14 8 5 1
Lower ssGSEA score 227 59 19 4 1 0
B
FCN1+S100A12+ Monocytes
Higher ssGSEA score
Lower ssGSEA score
Survival probability
p = 0.49
Time(years)
Number at risk
Higher ssGSEA score 254 67 15 6 4 0
Lower ssGSEA score 222 66 18 6 2 1
C
FCER1A+CD1C+ Dendritic cells
Higher ssGSEA score
Lower ssGSEA score
Survival probability
p = 0.0097
Time(years)
Number at risk
Higher ssGSEA score 228 69 16 7 4 1
Lower ssGSEA score 248 64 17 5 2 0
D
LILRA4+CLEC4C+ pDC
Higher ssGSEA score
Lower ssGSEA score
Survival probability
p = 0.0011
Time(years)
Number at risk
Higher ssGSEA score 247 79 16 8 5 1
Lower ssGSEA score 229 54 17 4 1 0
E
CLEC9A+XCR1+ cDC
Higher ssGSEA score
Lower ssGSEA score
Survival probability
p = 0.017
Time(years)
Number at risk
Higher ssGSEA score 233 79 16 9 5 1
Lower ssGSEA score 243 54 17 3 1 0
