# Supplementary material for: Single-cell RNA sequencing explores the evolution of the ecosystem from leukoplakia to head and neck squamous cell carcinoma
Source: Sci Rep. 2024 Apr 6;14:8097. doi: 10.1038/s41598-024-58978-9 (PMC10998855; doi:10.1038/s41598-024-58978-9)
Supplement: Supplementary file 7 — Supplementary Figure S6. [file 41598_2024_58978_MOESM7_ESM.pdf]

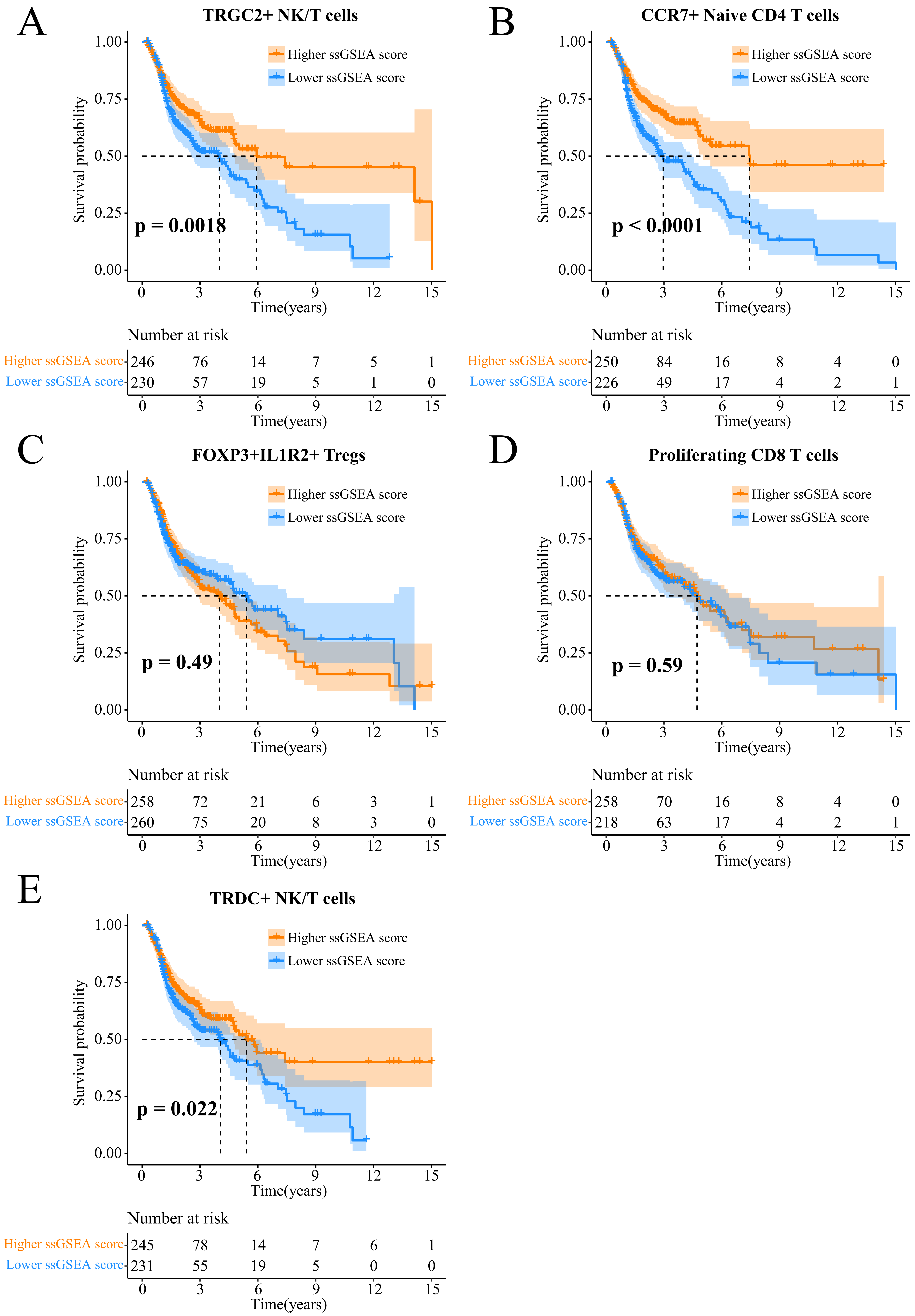
A
TRGC2+ NK/T cells
Higher ssGSEA score
Lower ssGSEA score
Survival probability
p = 0.0018
Time(years)
Number at risk
Higher ssGSEA score 246 76 14 7 5 1
Lower ssGSEA score 230 57 19 5 1 0
B
CCR7+ Naive CD4 T cells
Higher ssGSEA score
Lower ssGSEA score
Survival probability
p < 0.0001
Time(years)
Number at risk
Higher ssGSEA score 250 84 16 8 4 0
Lower ssGSEA score 226 49 17 4 2 1
C
FOXP3+IL1R2+ Tregs
Higher ssGSEA score
Lower ssGSEA score
Survival probability
p = 0.49
Time(years)
Number at risk
Higher ssGSEA score 258 72 21 6 3 1
Lower ssGSEA score 260 75 20 8 3 0
D
Proliferating CD8 T cells
Higher ssGSEA score
Lower ssGSEA score
Survival probability
p = 0.59
Time(years)
Number at risk
Higher ssGSEA score 258 70 16 8 4 0
Lower ssGSEA score 218 63 17 4 2 1
E
TRDC+ NK/T cells
Higher ssGSEA score
Lower ssGSEA score
Survival probability
p = 0.022
Time(years)
Number at risk
Higher ssGSEA score 245 78 14 7 6 1
Lower ssGSEA score 231 55 19 5 0 0
